# Performance of the GRACE 2.0 score in patients with type 1 and type 2 myocardial infarction

**DOI:** 10.1093/eurheartj/ehaa375

**Published:** 2020-06-09

**Authors:** John Hung, Andreas Roos, Erik Kadesjö, David A McAllister, Dorien M Kimenai, Anoop S V Shah, Atul Anand, Fiona E Strachan, Keith A A Fox, Nicholas L Mills, Andrew R Chapman, Martin J Holzmann

**Affiliations:** BHF Centre for Cardiovascular Science, University of Edinburgh, 49 Little France Crescent, EH16 4SB Edinburgh, UK; Department of Medicine, Karolinska Institute, 171 77 Solna, Stockholm, Sweden; Functional Area of Emergency Medicine, Karolinska University Hospital, 141 57 Huddinge, Stockholm, Sweden; Department of Medicine, Karolinska Institute, 171 77 Solna, Stockholm, Sweden; Functional Area of Emergency Medicine, Karolinska University Hospital, 141 57 Huddinge, Stockholm, Sweden; Institute of Health and Wellbeing, University of Glasgow, G12 8QQ Glasgow, UK; BHF Centre for Cardiovascular Science, University of Edinburgh, 49 Little France Crescent, EH16 4SB Edinburgh, UK; Usher Institute, University of Edinburgh, EH8 9AG Edinburgh, UK; CARIM School for Cardiovascular Diseases, Maastricht University, 6229 ER Maastricht, the Netherlands; Central Diagnostic Laboratory, Maastricht University Medical Center, 6229 ER Maastricht, the Netherlands; BHF Centre for Cardiovascular Science, University of Edinburgh, 49 Little France Crescent, EH16 4SB Edinburgh, UK; Usher Institute, University of Edinburgh, EH8 9AG Edinburgh, UK; BHF Centre for Cardiovascular Science, University of Edinburgh, 49 Little France Crescent, EH16 4SB Edinburgh, UK; BHF Centre for Cardiovascular Science, University of Edinburgh, 49 Little France Crescent, EH16 4SB Edinburgh, UK; BHF Centre for Cardiovascular Science, University of Edinburgh, 49 Little France Crescent, EH16 4SB Edinburgh, UK; BHF Centre for Cardiovascular Science, University of Edinburgh, 49 Little France Crescent, EH16 4SB Edinburgh, UK; Usher Institute, University of Edinburgh, EH8 9AG Edinburgh, UK; BHF Centre for Cardiovascular Science, University of Edinburgh, 49 Little France Crescent, EH16 4SB Edinburgh, UK; Department of Medicine, Karolinska Institute, 171 77 Solna, Stockholm, Sweden; Functional Area of Emergency Medicine, Karolinska University Hospital, 141 57 Huddinge, Stockholm, Sweden

**Keywords:** Type 1 myocardial infarction, Type 2 myocardial infarction, Universal definition, GRACE, High-sensitivity, Troponin

## Abstract

**Aims:**

The Global Registry of Acute Coronary Events (GRACE) score was developed to evaluate risk in patients with myocardial infarction. However, its performance in type 2 myocardial infarction is uncertain.

**Methods and results:**

In two cohorts of consecutive patients with suspected acute coronary syndrome from 10 hospitals in Scotland (*n* = 48 282) and a tertiary care hospital in Sweden (*n* = 22 589), we calculated the GRACE 2.0 score to estimate death at 1 year. Discrimination was evaluated by the area under the receiver operating curve (AUC), and compared for those with an adjudicated diagnosis of type 1 and type 2 myocardial infarction using DeLong’s test. Type 1 myocardial infarction was diagnosed in 4981 (10%) and 1080 (5%) patients in Scotland and Sweden, respectively. At 1 year, 720 (15%) and 112 (10%) patients died with an AUC for the GRACE 2.0 score of 0.83 [95% confidence interval (CI) 0.82–0.85] and 0.85 (95% CI 0.81–0.89). Type 2 myocardial infarction occurred in 1121 (2%) and 247 (1%) patients in Scotland and Sweden, respectively, with 258 (23%) and 57 (23%) deaths at 1 year. The AUC was 0.73 (95% CI 0.70–0.77) and 0.73 (95% CI 0.66–0.81) in type 2 myocardial infarction, which was lower than for type 1 myocardial infarction in both cohorts (*P* < 0.001 and *P* = 0.008, respectively).

**Conclusion:**

The GRACE 2.0 score provided good discrimination for all-cause death at 1 year in patients with type 1 myocardial infarction, and moderate discrimination for those with type 2 myocardial infarction.

**Trial registration:**

ClinicalTrials.gov number, NCT01852123.

**See page 2562 for the editorial comment on this article (doi: 10.1093/eurheartj/ehaa486)**

## Introduction

Coronary heart disease is responsible for around 2 million deaths across Europe every year.^1^ To improve prognostication and promote consistency in the investigation and management of patients with acute coronary syndrome, the Global Registry of Acute Coronary Events (GRACE) score was developed.^2–5^ The score applies clinical variables, the electrocardiogram, and cardiac biomarkers to estimate risk of future all-cause mortality and myocardial infarction. The use of the GRACE 2.0 score in patients with non-ST-segment elevation acute coronary syndrome has a class Ia recommendation for guiding prognosis and IIa recommendation for guiding management across all international guidelines.^6–8^

Since the introduction of the GRACE score, there have been significant changes in the way we diagnose myocardial infarction, driven by major improvements in the sensitivity of cardiac troponin. The Fourth Universal Definition of Myocardial Infarction recommends the use of high-sensitivity cardiac troponin (hs-cTn) assays and a sex-specific 99th centile diagnostic threshold for myocardial injury and infarction.^9^ These assays have the ability to quantify myocardial injury at a threshold 10-fold lower than was in use at the time of the original GRACE study. This increase in diagnostic sensitivity has led to an understanding that myocardial infarction can occur in a number of different clinical settings.^10–12^

The Fourth Universal Definition recognizes myocardial infarction may occur due to atheromatous plaque rupture and thrombosis (type 1 myocardial infarction), or secondary to an imbalance in myocardial oxygen supply or demand without coronary atherothrombosis (type 2 myocardial infarction).^9^ Patients with type 2 myocardial infarction are older and more often have comorbidities and are at higher risk of adverse outcomes with as few as 30% of patients alive at 5 years.^13^ Despite a significant increase in risk of non-cardiovascular death, patients with type 2 myocardial infarction appear to have a similar risk of future cardiovascular events as those with type 1 myocardial infarction.^14^ To date, there are no validated prognostic tools to estimate all-cause mortality or future cardiovascular events in this population. Our aim was to evaluate the performance of the GRACE 2.0 score for the prediction of all-cause death in patients with type 1 and type 2 myocardial infarction.

## Methods

### Study populations

We assessed the performance of the GRACE 2.0 score in two cohorts of consecutive patients presenting to the Emergency Department with suspected acute coronary syndrome in Scotland and in Sweden.

*High*-*S*ensitivity *T*roponin in the *E*valuation of patients with suspected *A*cute *C*oronary *S*yndrome (*High-STEACS*) is a stepped-wedge cluster randomized controlled trial to evaluate implementation of a hs-cTnI assay in consecutive patients with suspected acute coronary syndrome across 10 hospitals in Scotland.^15^ Troponin was measured using the Abbott ARCHITECT_*STAT*_ high-sensitive troponin I assay (Abbott Diagnostics, Chicago, IL, USA). This assay has an inter-assay coefficient of variation of <10% at 4.7 ng/L and a 99th centile of 16 ng/L in women and 34 ng/L in men.^16^ All patients attending the Emergency Department between June 2013 and March 2016 were identified as having suspected acute coronary syndrome by the attending clinician at the time troponin was requested, using an electronic form integrated into the clinical care pathway. Patients were excluded if they had been admitted previously during the trial period or were not resident in Scotland. We used regional and national registries to ensure complete follow-up for the trial population with outcome events adjudicated by a panel.

The study population from the Karolinska University Hospital in Stockholm was derived from an observational cohort study of all patients >25 years old with a visit to the Emergency Department with chest pain and at least one hs-cTn measurement from January 2011 to October 2014.^17^ ^,^ ^18^ Troponin was measured using the Roche Elecsys hs-cTnT assay (Roche Diagnostics, Mannheim, Germany). This assay has a limit of detection of 5 ng/L, and a limit of blank of 3 ng/L. The 99th percentile cut-off point is 14 ng/L, and the coefficient of variation is <10% at 13 ng/L. The hospital’s local administrative database was used to identify eligible patients. Patients were excluded if they had an estimated glomerular filtration rate of <15 mL/min/1.73 m^2^. The obtained data, together with laboratory data, were sent to the Swedish National Board of Health and Welfare who linked information on comorbidities and outcomes, use of medication, and dates and causes of death from the National Patient Register, the Prescribed Drug Register, and the Cause-of-Death register, respectively. The Patient Register has nationwide coverage on diagnoses at discharge and surgical procedures coded according to the International Classification of Disease. Details of study approvals are contained in the Supplementary material online.

### Adjudication of myocardial infarction and outcomes

All diagnoses were adjudicated in accordance with the Fourth Universal Definition of Myocardial Infarction.^9^ In both cohorts, two physicians independently reviewed all clinical information with discordant diagnoses resolved by a third reviewer (Scotland) or by consensus discussion (Sweden). Type 1 myocardial infarction was defined in those with suspected acute coronary syndrome with symptoms or signs of myocardial ischaemia on the electrocardiogram and evidence of myocardial necrosis: hs-cTnI concentration above the sex-specific 99th centile with a rise and/or fall in concentration where serial testing was performed (Scotland); hs-cTnT concentration above the uniform 99th centile with a delta of 3 ng/L (Sweden). Patients with myocardial necrosis, symptoms or signs of myocardial ischaemia, and evidence of increased myocardial oxygen demand or decreased supply secondary to an alternative condition without evidence of acute atherothrombosis were defined as type 2 myocardial infarction. Patients with hs-cTn concentrations above the 99th centile without symptoms or signs of myocardial ischaemia were classified as having myocardial injury. All non-ischaemic myocardial injury was classified as acute, unless a change of ≤20% was observed on serial testing,^9^ or the final adjudicated diagnosis was chronic heart failure or chronic renal failure, where the classification was chronic myocardial injury.

The primary outcome was all-cause death at 1 year, and the secondary outcome was all-cause death or type 1 myocardial infarction at 1 year. All in-hospital and community deaths are recorded on the National General Register of Scotland, and the Swedish Patient Register. Subsequent myocardial infarction events were identified through the electronic patient record in Scotland with adjudication as for the index diagnosis, and using ICD-10 coding (I21 and I22) from the Swedish Patient Register in the Swedish cohort.

### Statistical analysis

Baseline characteristics were summarized for each cohort and in groups by according to adjudicated diagnosis. Group-wise comparisons were performed using χ^2^, Kruskal–Wallis, or one-way analysis of variance tests as appropriate. We determined the GRACE 2.0 score for all-cause death, and for all-cause death or type 1 myocardial infarction at 1 year. This score includes age, heart rate, systolic blood pressure, creatinine as continuous variables, with categorical variables for Killip class, cardiac arrest at admission, ST-segment deviation, and elevated cardiac biomarkers (defined here as any hs-cTn concentration above the 99th centile). Where data were missing within the Scottish cohort, this was assumed to be at random, and we applied multiple imputation using chained equations with five imputations of the dataset. For imputation, we applied Bayesian linear regression models for continuous data (creatinine, heart rate, and systolic blood pressure), multinomial logistic regression for ordinal data (Killip class) and logistic regression for binary data (e.g. cardiac arrest status). We assessed overall GRACE 2.0 model discrimination by determining the area under the receiver operating curve (AUC) and compared performance in patients with type 1 and type 2 myocardial infarction using the DeLong method. We assessed model calibration both graphically, and by using the Hosmer–Lemeshow goodness of fit test. In addition, we assessed GRACE performance by evaluating previously defined categories of mortality risk (<3% low, ≥3 and ≤8% intermediate and >8% high risk) using the Kaplan–Meier method. We explored the impact of multiple imputation by performing a sensitivity analysis in which we evaluated the complete dataset only. These results were similar to the primary analysis and are presented in the Supplementary material online, *Table S1*. In *post hoc* analyses, we also determined performance of the GRACE 2.0 score for in-hospital death and of hs-cTn alone to predict all-cause death at 1 year. All analyses were performed in R (Version 3.5.1) and the code required to calculate the GRACE 2.0 score is available in Supplementary material online, Appendix *S*1.

## Results

### Study populations

The Scottish cohort consisted of 48 282 consecutive patients (61 ± 17 years, 47% women) with suspected acute coronary syndrome of whom 10 360 (21%) had hs-cTnI concentrations above the 99th centile. It was possible to adjudicate the diagnosis in 88% (9115/10 360) of patients. The final diagnosis was type 1 myocardial infarction in 55% (4981/9115), type 2 myocardial infarction in 12% (1121/9115), and acute or chronic myocardial injury in 18% (1676/9115) and 14% (1287/9115), respectively. The remainder of patients had type 4a (9/9115) or 4b (41/9115) myocardial infarction.

The Swedish cohort consisted of 22 589 consecutive patients with suspected acute coronary syndrome of whom 3853 (17%) patients had hs-cTnT concentrations above the 99th centile. The final diagnosis was type 1 myocardial infarction in 28% (1080/3853) of patients, and type 2 myocardial infarction in 6% (247/3853), with acute or chronic myocardial injury in 30% (1144/3853) and 35% (1347/3853), respectively.

### Patient characteristics

Compared to patients with a diagnosis of type 1 myocardial infarction, those with type 2 myocardial infarction were older (74 ± 14 vs. 68 ± 14 years), more likely to be women (55% vs. 40%), and more likely to have a history of cardiovascular disease. Similar differences were apparent in both cohorts (*Table 1*). Patients with type 2 myocardial infarction were less likely to be offered coronary angiography, revascularization or secondary prevention than those with type 1 myocardial infarction (*Table 2*). There were differences in the covariates which influence the GRACE 2.0 score between groups, with higher heart rates (101 vs. 77 b.p.m.), lower systolic blood pressures (130 vs. 141 mmHg) and a higher proportion of patients with increased Killip class observed in those with type 2 myocardial infarction (*Table 3*).

**Table 1:** Characteristics of the Scottish and Swedish cohorts of patients diagnosed with type 1 and type 2 myocardial infarction

	Scottish cohort			Swedish cohort		
	All patients	Type 1 myocardial infarction	Type 2 myocardial infarction	All patients	Type 1 myocardial infarction	Type 2 myocardial infarction
Number of participants	48 282	4981	1121	22 589	1080	247
Age (years), mean (SD)	61 (17)	68 (14)	74 (14)	56 (17)	69 (13)	72 (13)
Men, *n* (%)	25 720 (53)	2995 (60)	501 (45)	11 817 (52)	743 (69)	122 (49)
Past medical history, *n* (%)						
Myocardial infarction	4214 (9)	667 (13)	163 (15)	1885 (8)	184 (17)	47 (19)
Ischaemic heart disease	11 912 (25)	1519 (30)^a^	454 (40)	2570 (11)^b^	—	—
Cerebrovascular disease	2949 (6)	368 (7)	135 (12)	940 (4)	66 (6)	20 (8)
Diabetes mellitus	3518 (7)	802 (16)	147 (13)	2191 (10)	204 (19)	54 (22)
Heart failure hospitalization	4322 (9)	792 (16)	292 (26)	1244 (6)	86 (8)	40 (16)
Previous revascularization, *n* (%)						
Previous PCI or CABG	4464 (9)	592 (12)	129 (12)	1979 (9)	210 (19)	53 (22)
Medications at presentation, *n* (%)						
Aspirin	13 163 (27)	1694 (34)	471 (42)	4258 (19)	414 (38)	101 (41)
Statin	19 366 (40)	2377 (48)	632 (56)	4265 (19)	362 (34)	86 (35)
ACE inhibitor or ARB	15 618 (32)	1995 (40)	514 (46)	5547 (25)	456 (42)	130 (53)
Beta-blocker	13 173 (27)	1598 (32)	489 (44)	5508 (24)	441 (41)	140 (57)
Oral anti-coagulant^c^	3253 (7)	292 (6)	170 (15)	—	—	—
Electrocardiogram^d^						
Myocardial ischaemia	—	1872 (38)	383 (34)	—	281 (26)	67 (27)
Physiological parameters^d^						
Heart rate (b.p.m.)	—	79 (20)	105 (35)	—	76 (17)	94 (31)
Systolic blood pressure (mmHg)	—	142 (28)	132 (30)	—	153 (28)	142 (34)
Haematology and clinical chemistry						
Haemoglobin (g/L)	136 (22)	136 (22)	126 (29)	—	139 (17)	130 (20)
eGFR (mL/min)	54 (13)	51 (14)	46 (15)	88 (23)	74 (23)	66 (25)
Peak hs-cTnI (ng/L)	4 (2–16)	855 (104–6775)	125 (48–604)	—	—	—
Peak hs-cTnT (ng/L)	—	—	—	—	182 (49–616)	77 (32–173)

eGFR calculated according to the MDRD equation (mL/min).

a Defined as prior angina, myocardial infarction, or revascularization.

b Defined as prior myocardial infarction or revascularization.

^c,d^Electrocardiogram findings and physiological parameters provided for patients with myocardial infarction only.

**Table 2:** Rates of angiography, revascularization, and prescription for medical therapy on discharge in the Scottish and Swedish cohorts

	Scottish cohort		Swedish cohort	
	Type 1 myocardial infarction (*n* = 4981)	Type 2 myocardial infarction (*n* = 1121)	Type 1 myocardial infarction (*n* = 1064)	Type 2 myocardial infarction (*n* = 228)
Coronary angiography^a^	3083 (62)	123 (11)	—	—
PCI or CABG^a^	2217 (45)	24 (2)	543 (51)	7 (3)
Aspirin	3934 (79)	588 (52)	918 (86)	101 (44)
P2Y12 inhibitor	3544 (71)	319 (28)	871 (82)	26 (11)
ACE or ARB	3572 (72)	618 (55)	725 (68)	118 (52)
Beta-blocker	3476 (70)	708 (63)	169 (74)	946 (89)
Statin therapy	4141 (83)	700 (62)	883 (83)	92 (40)

Information from the Swedish cohort available in 99% (1064/1080) with type 1 and 92% (228/247) with type 2 myocardial infarction.

Values are number (%).

a Angiography and revascularization within 30 days of presentation *P* < 0.001 for all treatments in patients with type 1 vs. type 2 across both cohorts. *P*-values obtained from group-wise comparisons using χ^2^ test.

**Table 3:** Components of the GRACE 2.0 risk score in patients with type 1 and type 2 myocardial infarction

	Type 1 myocardial infarction		Type 2 myocardial infarction	
	Scottish cohort	Swedish cohort	Scottish cohort	Swedish cohort
Age	68 (14)	69 (13)	74 (14)	72 (13)
Heart rate (b.p.m.)	77 (65–92)	73 (64–84)	101 (81–125)	89 (72–109)
Systolic blood pressure (mmHg)	141 (123–160)	152 (135–170)	130 (111–150)	140 (120–160)
Creatinine	0.93 (0.80–1.19)	0.93 (0.79–1.13)	1.05 (0.82–1.39)	0.98 (0.78–1.27)
Killip class (%)				
I	4419 (88.7)	1014 (93.9)	852 (76)	188 (76.1)
II	279 (5.6)	64 (5.9)	126 (11.2)	51 (20.6)
III	205 (4.1)	2 (0.2)	130 (11.6)	8 (3.3)
IV	78 (1.6)	0 (0.0)	13 (1.6)	0 (0.0)
Cardiac arrest (%)	278 (5.6)	8 (0.1)	30 (2.7)	1 (0.4)
Troponin >99th centile at presentation (%)	4092 (82.2)	929 (86.0)	881 (78.6)	199 (80.6)
ECG ischaemia (%)	1627 (32.7)	281 (26.0)	311 (27.7)	67 (27.1)
STEMI (%)	915 (18.4)	0 (0)	5 (0.5)	0 (0)
GRACE 2.0 risk of death at 1 year (%)	4.9% (2.3–11.7%)	3.8% (1.9–8.2%)	11.2% (5.4–22.1%)	7.7% (3.5–19.9%)
GRACE 2.0 risk of death or MI at 1 year (%)	9.4% (5.6–18.2%)	7.7% (4.9–14.0%)	17.8% (10.2–29%)	13.3% (7.4–25.9%)

Values are represented as mean (SD) or median (IQR).

### GRACE risk score and prediction of death at 1 year

**Figure 1:**
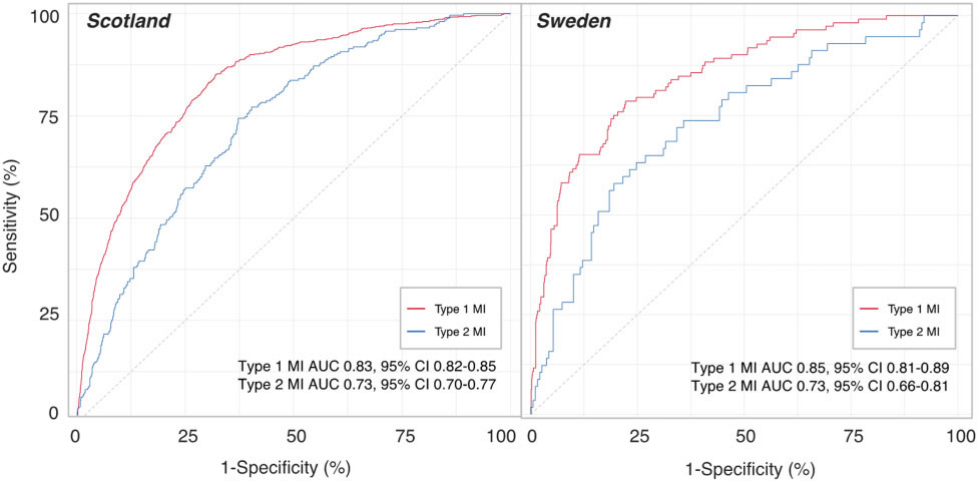
Comparison of the discrimination of the GRACE score for the prediction of all-cause mortality in patients with type 1 (red) and type 2 (blue) myocardial infarction using the area under the receiver operator characteristic curve, in the Scottish and Swedish cohorts.

We obtained follow-up in 100% of participants for primary and secondary outcomes at 1 year. In patients with type 1 myocardial infarction, 15% (720/4981) and 10% (112/1080) died from any cause at 1 year in the Scottish and Swedish cohorts, respectively. The GRACE 2.0 score was higher in those with type 2 compared to type 1 myocardial infarction across both cohorts (*Table 3*) and had good discriminative ability with an AUC of 0.83 [95% confidence interval (CI) 0.82–0.85] and 0.85 (95% CI 0.81–0.89), respectively (*Figure 1*).

In patients with type 2 myocardial infarction, 23% (258/1121) and 23% (57/247) died from any cause at 1 year in the Scottish and Swedish cohorts, respectively. The GRACE 2.0 score had moderate discriminative ability, with an AUC of 0.73 (95% CI 0.70–0.77) and 0.73 (95% CI 0.66–0.81), respectively, and performed less well than in patients with type 1 myocardial infarction (DeLong test, *P* < 0.001 and *P* = 0.008 vs. type 1 myocardial infarction in Scottish and Swedish cohorts, respectively, *Figure 1*).

**Figure 2:**
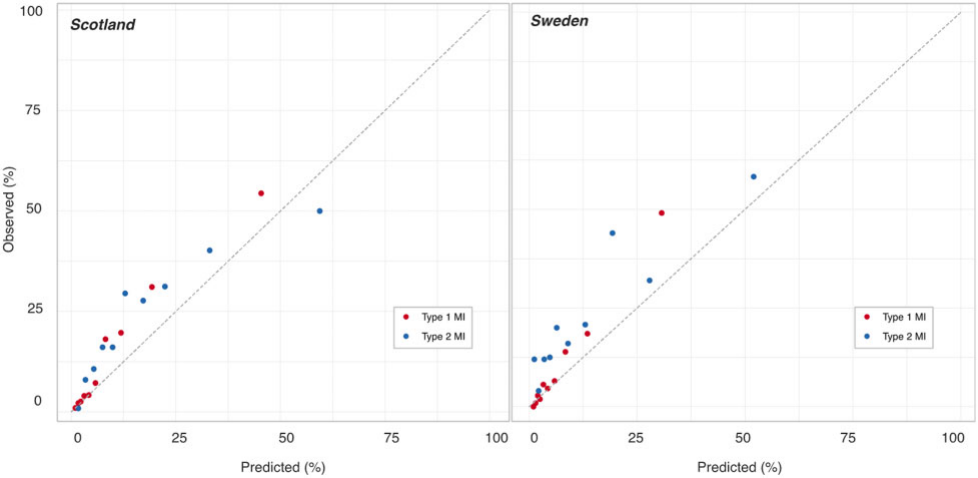
Evaluation of the calibration of the GRACE score for the prediction of all-cause mortality in patients with type 1 (red) and type 2 (blue) myocardial infarction, using the observed rate of events vs. the predicted rate of events, in the Scottish and Swedish cohorts. Each dot represents one decile of risk.

Similar performance was observed in men and women with type 1 and type 2 myocardial infarction in both cohorts (Supplementary material online, *Table S3*). Calibration plots and the Hosmer–Lemeshow test indicated the GRACE 2.0 score underestimated future all-cause death across all deciles of risk in both type 1 and type 2 myocardial infarction (*Figure 2*, Supplementary material online, *Table S2*).

### GRACE risk categories

**Figure 3:**
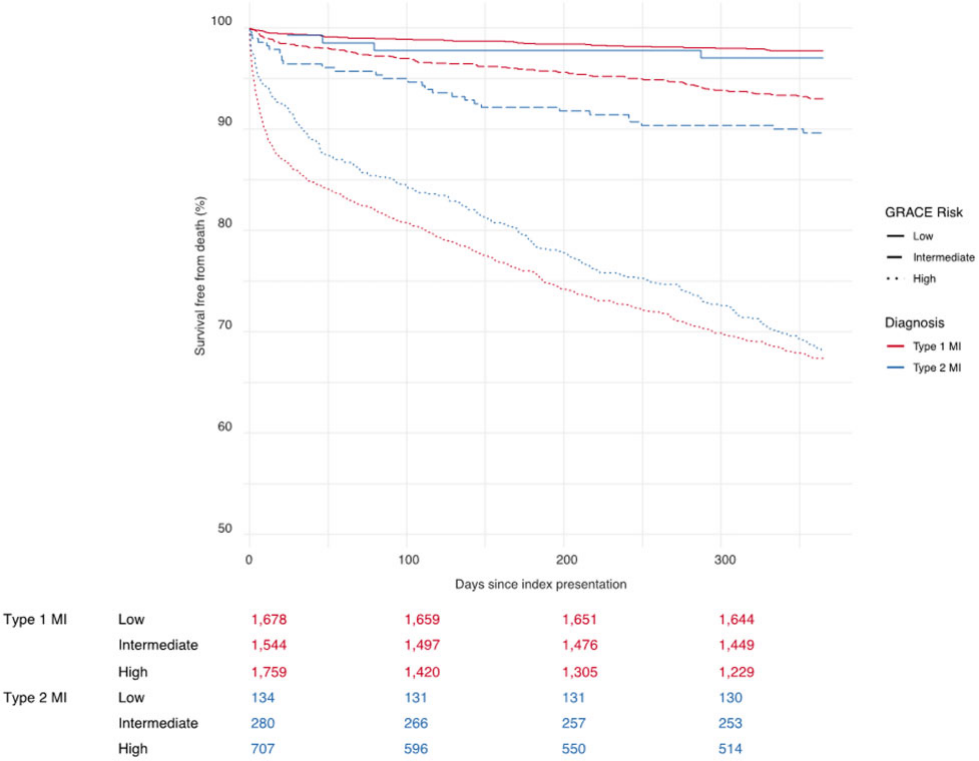
Comparison of survival free from death in patients with type 1 and type 2 myocardial infarction grouped by GRACE risk category (low risk <3%, green; intermediate risk ≥3 and <8%, orange; high risk ≥8%, red) in the Scottish cohort only.

In the Scottish cohort, we evaluated conventional GRACE categories of low, intermediate, and high predicted risk of mortality. Observed event rates were higher in patients with type 2 myocardial infarction who had a low or intermediate predicted risk of death (*Figure 3*). Baseline demographic information was similar across all categories of risk irrespective of the diagnosis of type 1 and type 2 myocardial infarction (Supplementary material online, *Table S4*). In patients with type 2 myocardial infarction in the Scottish cohort at low risk of death, there were fewer new prescription for aspirin (25% vs. 67%), statin (11% vs. 56%), angiotensin-converting enzyme inhibitor or angiotensin II receptor blocker (15% vs. 49%) compared to those with type 1 myocardial infarction*.* Similar patterns were observed in patients at intermediate or high risk of death.

**Take home figure:**
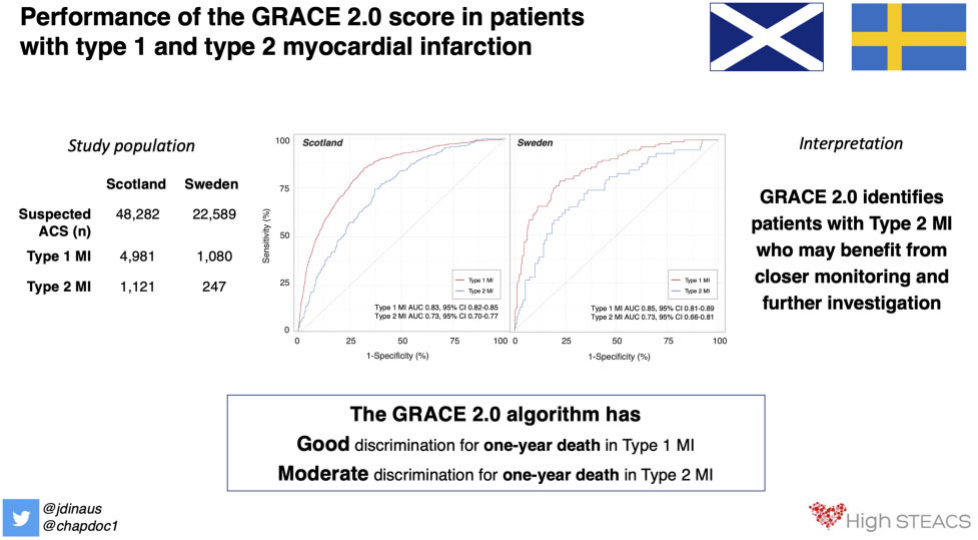
Performance of the GRACE 2.0 score in patients with type 1 and type 2 myocardial infarction.

### GRACE score and prediction of death or myocardial infarction

In patients with type 1 myocardial infarction, a total of 22% (1075/4981) and 16% (173/1080) of patients died or had a myocardial infarction at 1 year in the Scottish and Swedish cohorts, respectively. The AUC for the GRACE 2.0 model incorporating death or future myocardial infarction was 0.76 (95% CI 0.74–0.77) and 0.81 (95% CI 0.77–0.85), respectively.

In patients with type 2 myocardial infarction, there were 27% (297/1121) and 26% (63/247) deaths or myocardial infarctions at 1 year. Here, the GRACE 2.0 score gave an AUC of 0.70 (95% CI 0.67–0.74) and 0.72 (95% CI 0.65–0.80), respectively (*P* = 0.007 and *P* = 0.042 vs. type 1 myocardial infarction). Calibration plots showed the GRACE 2.0 model underestimated future risk in type 1 and type 2 myocardial infarction across both cohorts (Supplementary material online, *Figure S1*).

### *Post hoc* analysis

In a *post hoc* analysis, the GRACE 2.0 score had better discrimination for in-hospital death in patients with type 1 compared to type 2 myocardial infarction, where performance was moderate (Supplementary material online, *Table S5*). When applied as a continuous variable, both hs-cTnI and hs-cTnT were moderate predictors of all-cause death at 1 year (Supplementary material online, *Table S6*).

## Discussion

We evaluated the performance of the GRACE 2.0 score for the prediction of all-cause death, and all-cause death or myocardial infarction in consecutive patients with type 1 and type 2 myocardial infarction from two independent cohorts across two countries. We observe that the GRACE 2.0 score provides good discrimination for all-cause death in patients with type 1 myocardial infarction diagnosed using hs-cTn assays and for both cardiac troponin I and T. Consistent with the original validation study,^4^ discrimination for all-cause death was better than for death or myocardial infarction. In patients with type 2 myocardial infarction, the GRACE 2.0 score provided moderate discrimination in the prediction of all-cause death and performed less well in the prediction of all-cause death or myocardial infarction. As the GRACE 2.0 score performed better in patients with type 1 myocardial infarction, there may be opportunities to develop a bespoke model for risk prediction in patients with type 2 myocardial infarction.

The GRACE 2.0 score was derived prior to the publication of the first Universal Definition of Myocardial Infarction,^19^ which introduced a classification based on the underlying mechanism. Whilst type 1 myocardial infarction is caused exclusively by atherosclerotic plaque rupture and thrombotic coronary artery occlusion, type 2 myocardial infarction is a heterogeneous condition, occurring due to an imbalance in myocardial oxygen supply or an unmet need in myocardial oxygen demand in the context of another acute illness. A type 2 myocardial infarction may occur due to coronary pathology such as vasospasm, spontaneous dissection or coronary embolism, or with bystander stable coronary artery disease or normal coronary arteries in the context of tachyarrhythmia, severe hypoxia or hypotension. Whilst we have a strong evidence base for treatments which reduce all-cause mortality and future cardiovascular events in patients with type 1 myocardial infarction, at present, we have no guidelines to support investigation or management of patients with type 2 myocardial infarction, and in these patients clinical outcomes are worse, with as few as 30% of patients alive at 5 years.^20–25^

We recently demonstrated future cardiovascular risk was increased in all patients with myocardial injury and infarction, irrespective of diagnostic classification, despite a vast excess in non-cardiovascular death in patients without type 1 myocardial infarction. Patients with type 2 myocardial infarction were at almost four-fold increased risk of cardiovascular events relative to those without myocardial injury. This risk appears to be highest in those with a history of prior coronary artery disease, suggesting underlying coronary atheroma may at least in part be driving future cardiovascular risk.^26^ ^,^ ^27^ In order to identify patients with type 2 myocardial infarction who may benefit from further investigation and treatment, accurate risk stratification is required.

In this analysis, we demonstrate the GRACE 2.0 score performed well in the prediction of all-cause mortality and future cardiovascular events in patients with type 1 myocardial infarction, but discrimination was lower in those with type 2 myocardial infarction. At the time the GRACE was derived, the diagnosis of myocardial infarction was based on contemporary cardiac biomarkers with a diagnostic threshold at least 10-fold higher than advocated in current guidelines.^9^ The subsequent increase in sensitivity of cardiac troponin led to a reduction in the diagnostic threshold, and an increase in the recognition of myocardial injury and infarction in other conditions.^14^ A number of studies indicate a phenotypic distinction between patients with type 1 and type 2 myocardial infarction.^14^ ^,^ ^20–28^ Those with type 2 myocardial infarction are older, more often female, with lower haemoglobin and impairment in renal function. The GRACE 2.0 score was not derived in these patients, and although we found its performance to be acceptable with an AUC of 0.72 for all-cause death, it is perhaps not surprising it performed less well when compared with type 1 myocardial infarction.

Some attempts have been made to derive risk stratification tools in patients with type 2 myocardial infarction. The TARRACO risk score was derived in 611 patients with type 2 myocardial infarction and myocardial injury.^27^ This score applies troponin concentrations from a contemporary sensitive assay and the covariates age, hypertension, dyspnoea, anaemia, and the absence of chest pain and had moderate discrimination for future major adverse cardiovascular events (AUC 0.74, 95% CI 0.70–0.79). However, in a recent direct comparison of the GRACE, TIMI, and TARRACO scores in 359 patients with type 2 myocardial infarction from a single tertiary cardiac centre, only the GRACE score was predictive of all-cause mortality at 90 days (AUC 0.70, 95% CI 0.63–0.77), performing better than the bespoke TARRACO score (AUC 0.52, 95% CI 0.46–0.58).^28^

Analysis of the calibration of the GRACE 2.0 model in type 1 and type 2 myocardial infarction identified underestimation of risk across all outcomes. This likely reflects differences between the population of consented patients recruited into the GRACE registry, and the consecutive patient cohort evaluated here. There are a number of potentially important comorbidities not included in the GRACE 2.0 score which are common in clinical practice and could influence survival, particularly in those with type 2 myocardial infarction. These include atrial fibrillation, chronic obstructive pulmonary disease, heart failure, malignancy, dementia, and frailty. Furthermore, as suggested in a *post hoc* analysis, incorporating troponin concentration as a continuous variable could offer improved performance. Whether the inclusion of absolute troponin concentration, comorbidities or additional covariates, such as haemoglobin concentration, could improve model performance requires exploration.

Given the implementation of hs-cTn assays has been shown to increase recognition and the prevalence of type 2 myocardial infarction, there is an urgent and unmet need to improve risk prediction in these patients. Until bespoke risk prediction tools are available for patients with type 2 myocardial infarction, the GRACE 2.0 allows identification of patients at increased risk of death, both in-hospital and at 1 year. This may be helpful to guide clinicians when reviewing patients with type 2 myocardial infarction and deciding who may benefit from more intensive monitoring or further investigation for underlying coronary disease. We observed lower prescription rates for secondary prevention therapy in patients with type 2 myocardial infarction. This was most evident in those classified by GRACE as low- or intermediate risk, where rates of prescriptions for new antiplatelet or statin therapies in type 2 myocardial infarction were half those of type 1 myocardial infarction, and outcomes were worse. In those classified as high risk, prescription rates and outcomes between patients with type 1 and type 2 myocardial infarction were similar. Whether secondary prevention therapy in patients with type 2 myocardial infarction will improve clinical outcomes requires evaluation in prospective trials.^14^

We acknowledge some limitations. Firstly, whilst the GRACE 2.0 score was derived and validated across 14 countries, we only evaluate performance in Scotland and Sweden. However, we included consecutive patients across two different healthcare systems using different high-sensitivity troponin assays and found consistent results. The consistency in results was evident despite differences in the original study design and in the selection of patients between the two healthcare sites. Second, whilst we adjudicated all diagnoses according to the latest Fourth Universal Definition of Myocardial Infarction using all available clinical information, we acknowledge that diagnostic misclassification is possible. In the Scottish cohort where there was consensus amongst the adjudication panel that there was insufficient clinical information to make a definitive diagnosis, because of missing admission or discharge letters, we did not attempt to adjudicate the diagnosis. Third, where information on covariates required for calculating the GRACE score was missing this was determined to be at random, and to minimize bias we applied multiple imputation. As data were missing in patients with type 1 and type 2 myocardial infarction in equal proportion, and we observed consistent performance in a sensitivity analysis restricted to the dataset where complete case data were available, we do not think this impacted on the results observed. Fourth, we acknowledge that our analysis of in-hospital events was *post hoc* and is limited by a small number of events. Finally, we acknowledge that the rates of coronary angiography were lower here than in other registries or clinical trials of selected patient populations.^29^ ^,^ ^30^ We enrolled all consecutive patients in both cohorts, where older patients with comorbidities managed out with the coronary care unit were included rather than excluded. This improves the generalizability of our findings, and whilst angiography is not required for the diagnosis of myocardial infarction, lower rates may have contributed to diagnostic misclassification and influenced performance of the GRACE 2.0 score.

The GRACE 2.0 score provided good discrimination for all-cause death at 1 year in patients with type 1 myocardial infarction, and moderate discrimination for those with type 2 myocardial infarction. Until specific risk prediction tools are derived and validated, clinicians should consider applying the GRACE 2.0 score to guide prognosis and subsequent management in patients with type 2 myocardial infarction.

## Supplementary material

Supplementary material is available at *European Heart Journal* online.

## Funding

This trial was funded by a Special Project Grant from the British Heart Foundation (BHF) (SP/12/10/29922) with additional support from a BHF Research Excellence Award (RE/18/5/34216). A.R.C. and N.L.M. are supported by Clinical Research Training Fellowship (FS/16/75/32533) and the Butler Senior Clinical Research Fellowship (FS/16/14/32023) from the BHF. A.R.C. is supported by a Starter Grant for Clinical Lecturers from the Academy of Medical Sciences, which is supported by the Wellcome Trust, the Medical Research Council, the British Heart Foundation, vs. Arthritis, Diabetes UK, and the British Thoracic Society [SGL021/1075]. A.R. holds a research position funded by the regional agreement on medical training and clinical research between Stockholm County Council and Karolinska Institutet (20160644). D.M.K. was supported by a grant from Health Data Research UK which receives its funding from HDR UK Ltd (HDR-5012) funded by the UK Medical Research Council, Engineering and Physical Sciences Research Council, Economic and Social Research Council, Department of Health and Social Care (England), Chief Scientist Office of the Scottish Government Health and Social Care Directorates, Health and Social Care Research and Development Division (Welsh Government), Public Health Agency (Northern Ireland), British Heart Foundation, and the Wellcome Trust. M.J.H. holds research positions funded by the Swedish Heart-Lung Foundation (20170804) and the Stockholm County Council (20170686). Abbott Laboratories provided cardiac troponin assay reagents, calibrators, and controls without charge for the High-STEACS trial. The funders played no role in the design, conduct, data collection, analysis or reporting of the trial.

**Conflict of interest:** A.S.V.S. and A.R.C. have received honoraria from Abbott Diagnostics. N.L.M. has received honoraria from Abbott Diagnostics, Siemens Healthineers, Roche Diagnostics and Singulex, and the University of Edinburgh has received research grants from Abbott Diagnostics and Siemens Healthineers. M.J.H. has received consultancy honoraria from Idorsia.

## Supplementary Material

ehaa375_Supplementary_Data
