# Supplementary material for: Performance of the GRACE 2.0 score in patients with type 1 and type 2 myocardial infarction
Source: Eur Heart J. 2020 Jun 9;42(26):2552–61. doi: 10.1093/eurheartj/ehaa375 (PMC8266602; doi:10.1093/eurheartj/ehaa375)
Supplement: ehaa375_Supplementary_Data [file ehaa375_supplementary_data.pdf]

SUPPLEMENTARY DATA

Performance of the GRACE 2.0 score in patients with type 1 and type 2 myocardial infarction

John Hung, M.D.[1*], Andreas Roos, M.D. Ph.D.[2,3*], Erik Kadesjö, M.D.[2,3], David A. McAllister, M.D.[4], Dorien M. Kimenai, Ph.D.[1,5-7] Anoop S.V. Shah, M.D. Ph.D.[1,5], Atul Anand, M.D Ph.D.[1], Fiona E Strachan, Ph.D[1], Keith A.A. Fox M.D.[1], Nicholas L. Mills, M.D. Ph.D.[1,5], Andrew R. Chapman, M.D. Ph.D.[1*], Martin J. Holzmann, M.D. Ph.D.[2,3*]

[1] BHF Centre for Cardiovascular Science, University of Edinburgh, Edinburgh, UK
[2] Department of Medicine, Karolinska Institute, Solna, Stockholm, Sweden
[3] Functional Area of Emergency Medicine, Karolinska University Hospital, Huddinge, Stockholm, Sweden
[4] Institute of Health and Wellbeing, University of Glasgow, Glasgow, UK
[5] Usher Institute, University of Edinburgh, Edinburgh, UK
[6] CARIM School for Cardiovascular Diseases, Maastricht University, Maastricht, the Netherlands
[7] Central Diagnostic laboratory, Maastricht University Medical Center, Maastricht, the Netherlands

[*]These authors contributed equally

Corresponding Author:
Dr Andrew R Chapman
BHF/University Centre for Cardiovascular Science
The University of Edinburgh
Edinburgh EH16 4SA
United Kingdom
Telephone: +44 131 242 6515
Fax: +44 131 242 6379
E-mail: a.r.chapman@ed.ac.uk

Tables and Figures: 6

Study Approvals

The High-STEACS trial was approved by the Scotland A Research Ethics Committee, the Public Benefit and Privacy Panel for Health and Social Care, and by each National Health Service (NHS) Health Board. All data were collected prospectively from the electronic patient record, deidentified and linked within secure NHS Safe Havens. In the Karolinska University Hospital cohort, the study protocol was approved by the Regional Ethical Review Board in Stockholm. Both studies were conducted in accordance with the Declaration of Helsinki.

Table S1. Performance of the GRACE 2.0 score for death and death or myocardial infarction in the Scottish cohort with and without multiple imputation.

Scottish cohort

	**Type 1 MI**		**Type 2 MI**	
	**Complete only (n=2,538)**	**With imputation (n=4,981)**	**Complete only (n=642)**	**With imputation (n=1,121)**
**Death**	378 (15%)	720 (15%)	144 (23%)	258 (23%)
**AUC for Death (c-statistic)**	0.85 (0.83-0.87)	0.83 (0.82-0.85)	0.71 (0.66-0.75)	0.73 (0.70-0.77)
**Death or MI**	560 (22%)	1,075 (22%)	166 (26%)	297 (27%)
**AUC for Death or MI (c-statistic)**	0.78 (0.76-0.80)	0.76 (0.74-0.77)	0.69 (0.64-0.74)	0.70 (0.67-0.74)

AUC: area under the receiver-operator-curve; HL: Hosmer-Lemeshow test.

As we used electronic records and enrolled consecutive patients into the High-STEACS trial, some variables required for the calculation of the GRACE score were missing from our dataset. This was most commonly due to the omission of routine observations which was assumed to be at random. To maximise the available dataset and to minimise bias from excluding participants, we applied multiple imputation using chained equations with five imputations of the dataset, using the *mice* package in R. For imputation we applied Bayesian linear regression for continuous data (creatinine, heart rate, systolic blood pressure), multinomial logistic regression for ordinal data (Killip class) and logistic regression for binary data (cardiac arrest status). Data was missing for the following variables (n,%) ; creatinine (64, 1%), cardiac arrest status (460, 7.5%), ECG ischaemia (718, 11.8%), Killip class (1,079, 17.7%), heart rate (1,360, 22.3%) and systolic blood pressure (2,444, 40.1%).

Table S2. Performance of the GRACE 2.0 score for all-cause death and all-cause death or myocardial infarction at one year in the Scottish and Swedish cohorts

	Type 1 myocardial infarction		Type 2 myocardial infarction	
	**Scottish cohort (n=4,981)**	**Swedish cohort (n=1,080)**	**Scottish cohort (n=1,121)**	**Swedish cohort (n=247)**
**All-cause death**	720 (15%)	112 (10%)	258 (23%)	57 (23%)
**AUC (95% CI)**	0.83 (0.82-0.85)	0.85 (0.81-0.89)	0.73 (0.70-0.77)	0.73 (0.66-0.81)
**$\chi 2$ and P-value for HL**	159.4 <0.001	27.3 <0.001	77.9 <0.001	54.2 <0.001
**P-value for DeLong test**	Reference	Reference	<0.001	0.008
**All-cause death or MI**	1,075 (22%)	173 (16%)	297 (27%)	63 (26%)
**AUC (95% CI)**	0.76 (0.74-0.77)	0.81 (0.77-0.85)	0.70 (0.67-0.74)	0.72 (0.65-0.80)
**P-value for HL**	244.5 <0.001	52.9 <0.001	46.6 <0.001	14.8 0.064
**P-value for DeLong test**	Reference	Reference	0.007	0.042

AUC: area under the receiver-operator-curve; CI = confidence interval, HL: Hosmer-Lemeshow test. The principle of the Hosmer-Lemeshow test is to compare the concordance between predicted and actual event rates. Based on the predicted probability, the data is divided into ten groups. In each of these groups the predicted and actual are calculated, and a $\chi 2$ statistic is calculated to compare the differences between predicted and actual event rates (sum of [actual-expected]2/expended). Small $\chi 2$ values with a p-value close to 1 indicates a good calibration

Table S3. Performance of the GRACE 2.0 score for all cause death and death or myocardial infarction by sex

	**Type 1 myocardial infarction**				**Type 2 myocardial infarction**			
	**Scottish cohort**		**Swedish cohort**		**Scottish cohort**		**Swedish cohort**	
	**Men (n=2,995)**	**Women (n=1,986)**	**Men (n=743)**	**Women (n=337)**	**Men (n=501)**	**Women (n=620)**	**Men (n=122)**	**Women (n=125)**
**All-cause death AUC (95% CI)**	0.85 (0.83-0.87)	0.81 (0.79-0.84)	0.85 (0.81-0.90)	0.84 (0.77-0.90)	0.74 (0.69-0.78)	0.73 (0.69-0.77)	0.74 (0.65-0.84)	0.72 (0.60-0.84)
**P-value for DeLong test (Men versus Women)**	0.04		0.70		0.86		0.77	
**All-cause death or MI AUC (95% CI)**	0.76 (0.74-0.78)	0.74 (0.72-0.77)	0.81 (0.77-0.86)	0.79 (0.73-0.86)	0.71 (0.66-0.76)	0.70 (0.65-0.74)	0.76 (0.66-0.85)	0.69 (0.57-0.80)
**P-value for DeLong test**	0.23		0.61		0.59		0.36	

Table S4. Characteristics of Scottish cohort stratified by low, intermediate and high GRACE risk categories.

	Low Risk GRACE (<3%)		Intermediate Risk GRACE (≥3 and ≤8%)		High Risk GRACE (>8%)	
	**Type 1 MI**	**Type 2 MI**	**Type 1 MI**	**Type 2 MI**	**Type 1 MI**	**Type 2 MI**
No. of participants (%)	1,826 (37)	131 (12)	1,511 (30)	305 (27)	1,644 (33)	685 (61)
Age (years), mean (SD)	55 (9)	51 (12)	70 (9)	68 (10)	80 (10)	81 (9)
Men, n (%)	1281 (70)	54 (41)	861 (57)	156 (51)	853 (52)	291 (42)
Past medical history						
Myocardial infarction, n (%)	194 (11)	6 (5)	188 (12)	45 (15)	285 (17)	112 (16)
Ischemic heart disease, n (%)	335 (18)	12 (9)	485 (32)	111 (36)	699 (43)	331 (48)
Cerebrovascular disease, n (%)	49 (3)	5 (4)	97 (6)	23 (8)	222 (14)	107 (16)
Diabetes mellitus, n (%)	205 (11)	6 (5)	264 (17)	35 (11)	333 (20)	106 (15)
Heart failure hospitalisation, n (%)	89 (5)	6 (5)	207 (14)	61 (20)	496 (30)	225 (33)
New medication						
Aspirin, n (%)	1217 (67)	33 (25)	635 (42)	40 (13)	388 (24)	44 (6)
DAPT, n (%)	1409 (77)	20 (15)	928 (61)	35 (11)	632 (38)	61 (9)
Statin, n (%)	1020 (56)	15 (11)	501 (33)	27 (9)	243 (15)	26 (4)
ACE inhibitor or ARB, n (%)	886 (49)	20 (15)	473 (31)	33 (11)	218 (13)	51 (7)
Beta-blocker, n (%)	961 (53)	39 (30)	560 (37)	74 (24)	357 (22)	106 (15)
Oral anti-coagulant, n (%)‡	18 (1)	23 (18)	55 (4)	62 (20)	56 (3)	124 (18)
Electrocardiogram§						
Myocardial ischemia	671 (37)	35 (27)	551 (36)	103 (34)	650 (40)	245 (36)
Physiological parameters§						
Heart rate, beats per minute	74 (16)	93 (36)	77 (19)	108 (40)	86 (21)	105 (32)
Systolic blood pressure, mmHg	149 (25)	148 (26)	142 (26)	140 (29)	134 (30)	126 (29)
Hematology and clinical chemistry						
Haemoglobin, g/L	146 (18)	138 (24)	136 (21)	132 (30)	126 (23)	120 (29)
eGFR, ml/min	59 (7)	56 (12)	54 (12)	54 (11)	43 (15)	42 (15)
Peak hs-cTn, ng/L	808 [132, 6255]	111 [44, 513]	787 [92, 5437]	128 [45, 672]	1063 [93, 8626]	124 [51, 609]

Table S5. Performance of GRACE 2.0 for the prediction of in-hospital death in the Scottish and the Swedish cohorts

	**Scottish cohort**		**Swedish cohort**	
	**Type 1 MI (n=4,981)**	**Type 2 MI (n=1,121)**	**Type 1 MI (n=1,080)**	**Type 2 MI (n=247)**
**In-hospital death n (%)**	74 (1.5)	25 (2.2)	27 (2.5)	14 (5.7)
**AUC (95% CI)**	0.85 (0.81-0.89)	0.67 (0.57-0.78)	0.85 (0.78-0.93)	0.82 (0.70-0.94)
**$\chi 2$ and P-value for HL**	100<br><0.001	50<br><0.001	26<br>0.01	444<br><0.001
**P value for DeLong test**	0.002		0.65	

Table S6. Performance of high-sensitivity cardiac troponin assays alone for the prediction of death at one year

	**Scottish cohort**		**Swedish cohort**	
	**Type 1 MI (n=4,981)**	**Type 2 MI (n=1,121)**	**Type 1 MI (n=1,080)**	**Type 2 MI (n=247)**
**Death at one year AUC (95% CI)**	0.58 (0.56-0.61)	0.62 (0.58-0.65)	0.64 (0.58-0.69)	0.72 (0.65-0.80)
**P value for DeLong test**	0.18		0.65	

Figure S1. Observed versus predicted all-cause mortality or myocardial infarction events in type 1 and type 2 myocardial infarction according to the GRACE 2.0 algorithm in the Scottish and Swedish cohorts.

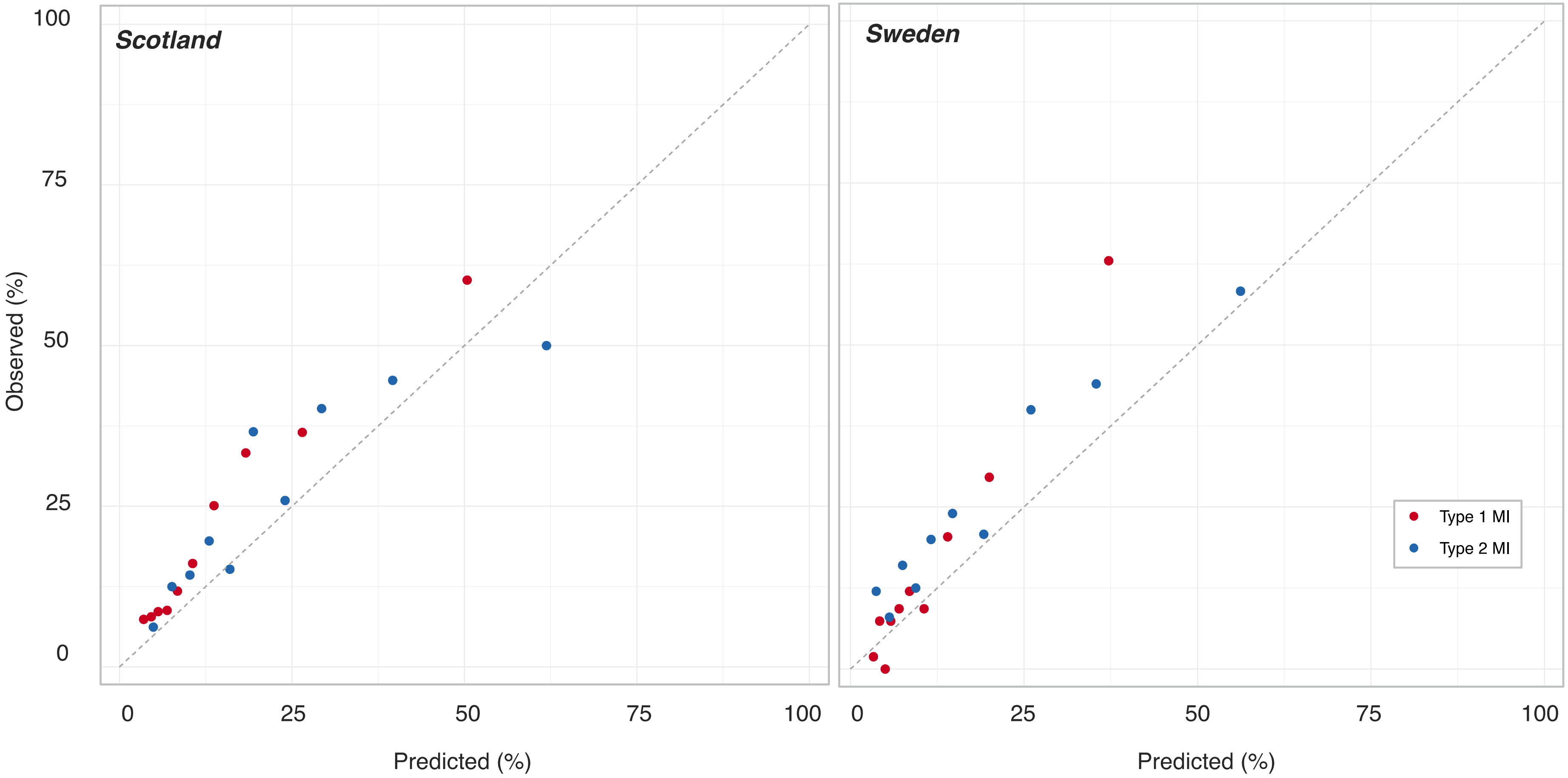

Supplementary Appendix

The R code required for the calculation of GRACE 2.0 coefficients for the estimation of in-hospital or one-year death, and one-year death or myocardial infarction will be made available on publication in the GitHub repository at the below address.

https://github.com/a-r-chapman
